# Supplementary material for: Prioritising Climate Change Mitigation Behaviours and Exploring Public Health Co-Benefits: A Delphi Study
Source: Int J Environ Res Public Health. 2023 Mar 14;20(6):5094. doi: 10.3390/ijerph20065094 (PMC10049208; doi:10.3390/ijerph20065094)
Supplement: Supplementary file 1 [file ijerph-20-05094-s001.zip › Supplementary Materials.pdf]

SUPPLEMENTARY MATERIALS

S1. Round 1: 37 climate change mitigation behaviours

Heat

- Install double/triple glazing
- Install cavity wall insulation
- Connect to District Heat Network (DHN)
- Install solid wall insulation
- Install Thermostatic Radiator Valves (TRVs)
- Take up a service-based heat proposition
- Reduce thermostat temperature
- Reduce number of rooms heated
- Heat for fewer hours of the day
- Cooling house by opening windows
- Rinsing the dishes and washing hands in cold water
- Cooking shorter meals or meals in bulk

Transport

- Reduce number of air miles
- Plug in electric vehicle (EV) whenever possible and accept smart charging
- Walk or cycle to school
- Buying and using a smaller car
- Reducing number of cars per household
- Combining trips
- Living closer to work and amenities
- Fewer holidays
- Extending how long a car is used for

Electricity

- Install LED lighting

- Buy smart-ready appliances
- Switch to time-of-use tariff
- Charging EV at home (smart charging, V2G, etc)
- Wait for a full load before using washing machine
- Doing dishes by hand
- Buying a smaller refrigerator

Consumption

- Reducing waste food
- Buy sustainable products
- Consumers buy on strength of companies/manufacturers carbon footprint
- Eat healthy/eat more fruit and veg

Non-sectoral/Societal Change

- Improving home workspace
- Transition to digital working
- Looking for jobs in a new green economy
- Looking for jobs closer to home
- Informed property purchasing

S2. Target behaviour discussion questions

Questions

- If you were able to conduct research on these behaviours for a year, which behaviour/behaviours would you want to focus resources on?
    - *Why is the behaviour important?*
    - *What do you think the relationship is between climate change behaviours and health-related impacts?*
    - What are the barriers and facilitators for the behaviour?

- If you were able to conduct research on these behaviours for a year what would you like to see happen?

- What existing work are you aware of?
    - *Can you identify any existing interventions that address the target behaviours?*
    - *What are the key outcomes of interventions?*
    - *What interventions haven't been tested/evaluated?*
    - *Can we identify/assess an intervention's capacity to produce long-term behaviour change?*
